# Supplementary material for: UHPLC-Orbitrap-MS Tentative Identification of 51 Oleraceins (Cyclo-Dopa Amides) in Portulaca oleracea L. Cluster Analysis and MS2 Filtering by Mass Difference
Source: Plants (Basel). 2021 Sep 15;10(9):1921. doi: 10.3390/plants10091921 (PMC8473048; doi:10.3390/plants10091921)
Supplement: Supplementary file 1 [file plants-10-01921-s001.zip › Figures S1-5, Table S7.pdf]

Figure S1. Estimating the optimal number of clusters with *k-means* clustering

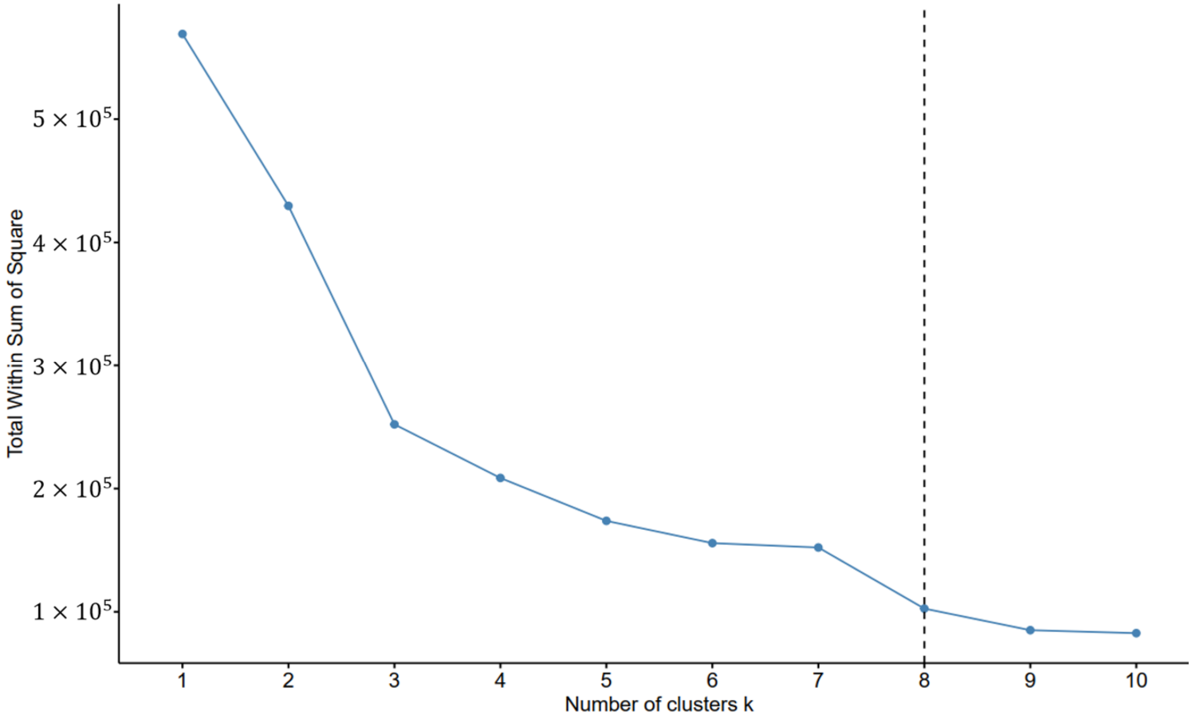

Figure S2. Estimating the optimal number of clusters with *pam* clustering

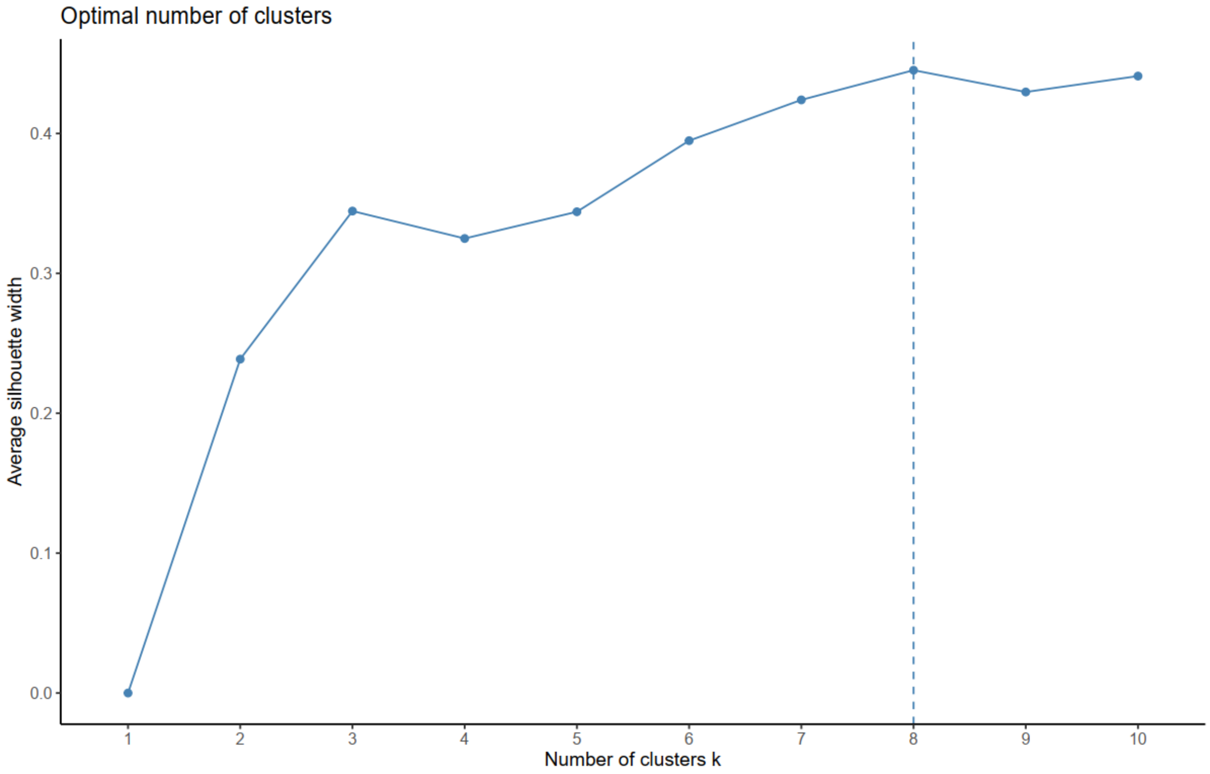

Figure S3. Scree plot representing the percentage of variances explained by each principal component

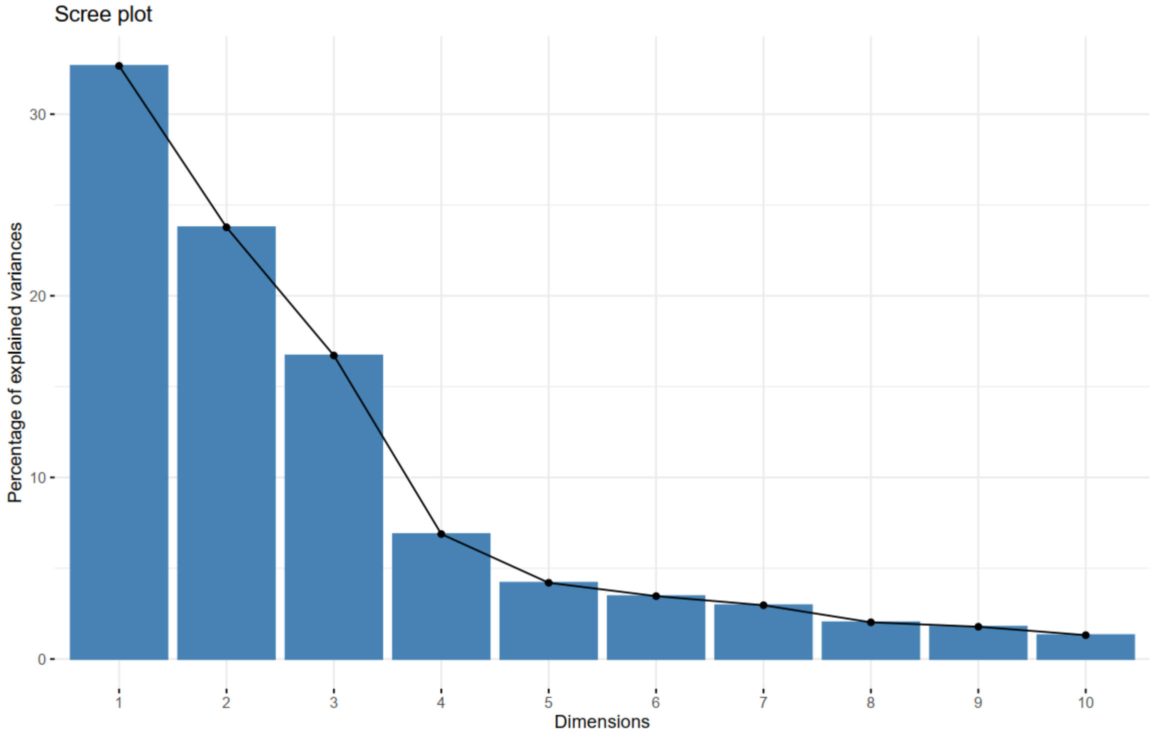

Figure S4. Visualization of the quality of representation of individuals (cos2)

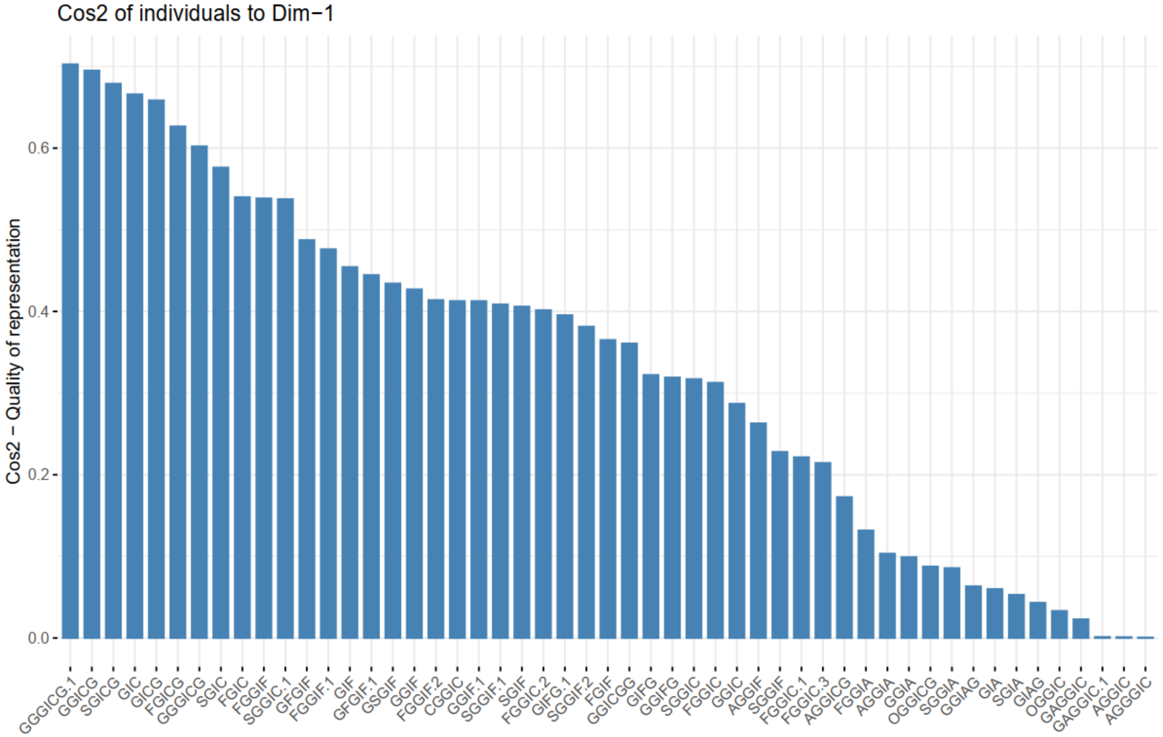

Figure S5. Visualization of the contribution of individuals on PC1 and PC2

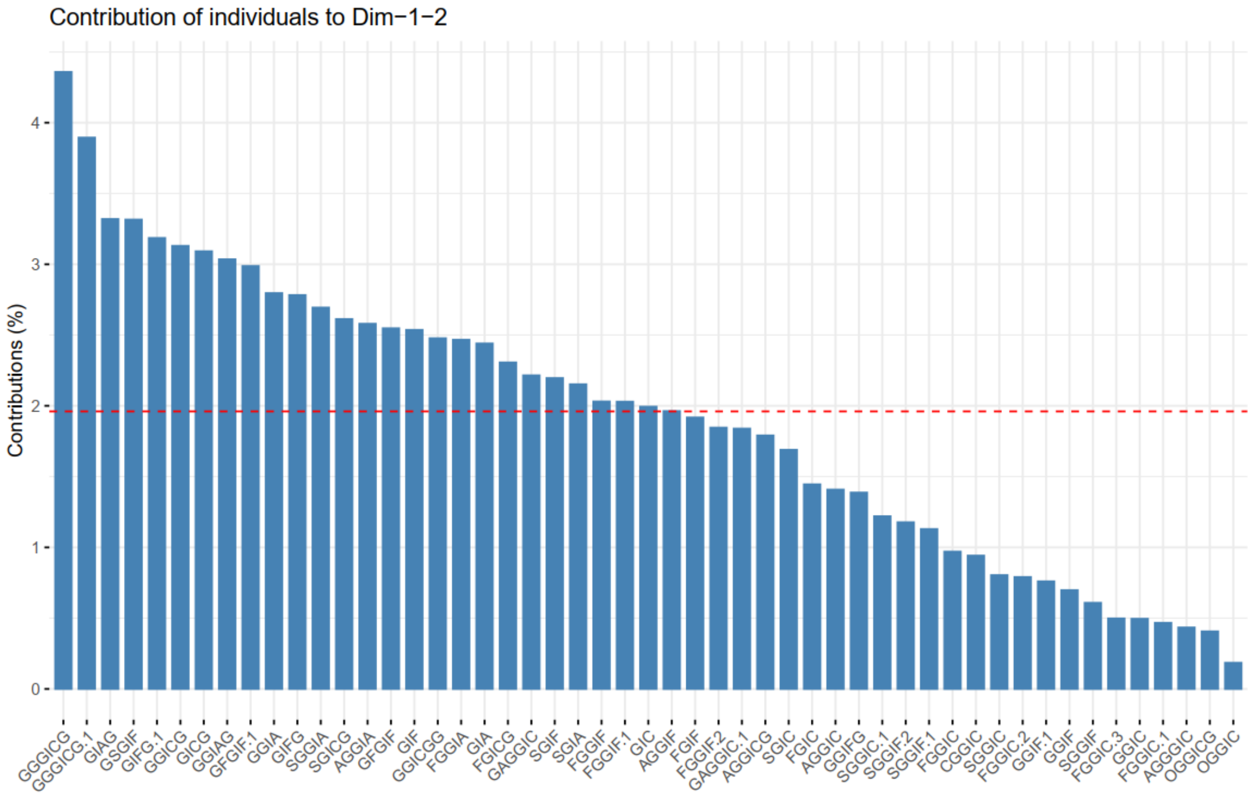

Table S7. Representative raw HR-Orbitrap-MS^2 spectra of the 51 identified oleraceins in negative ionization mode. For list of used abbreviations see “4.9. Used abbreviations” in the manuscript.

GIC

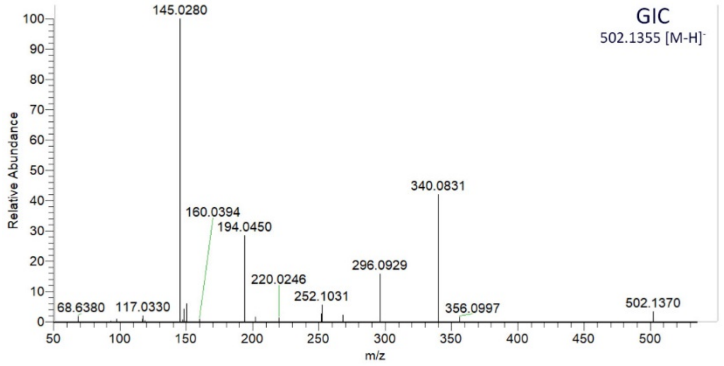

GIA

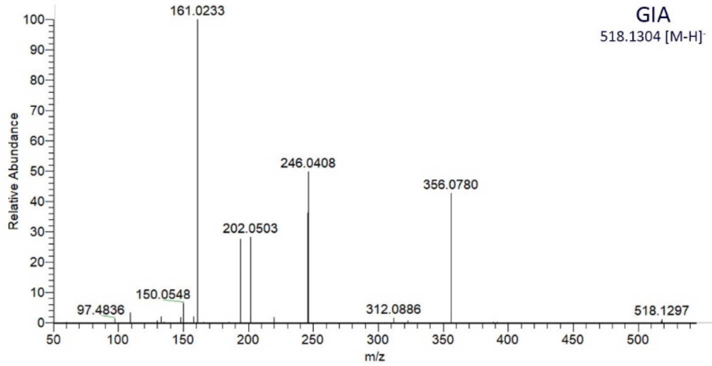

GICG

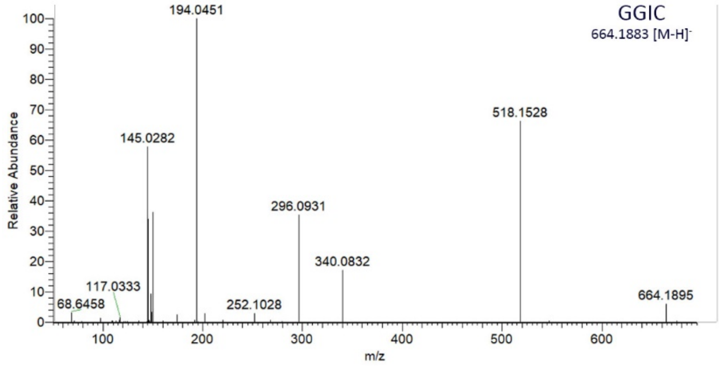

GGIC

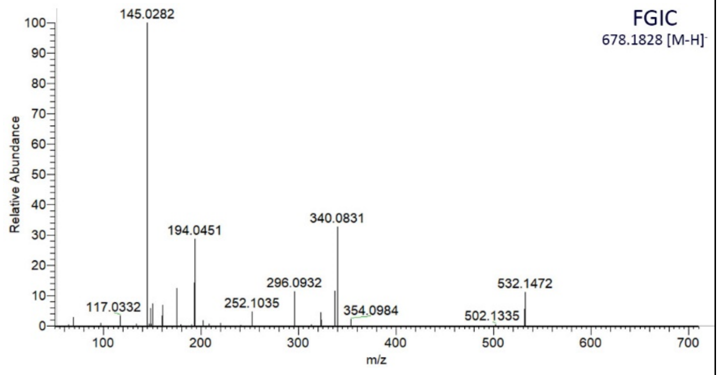

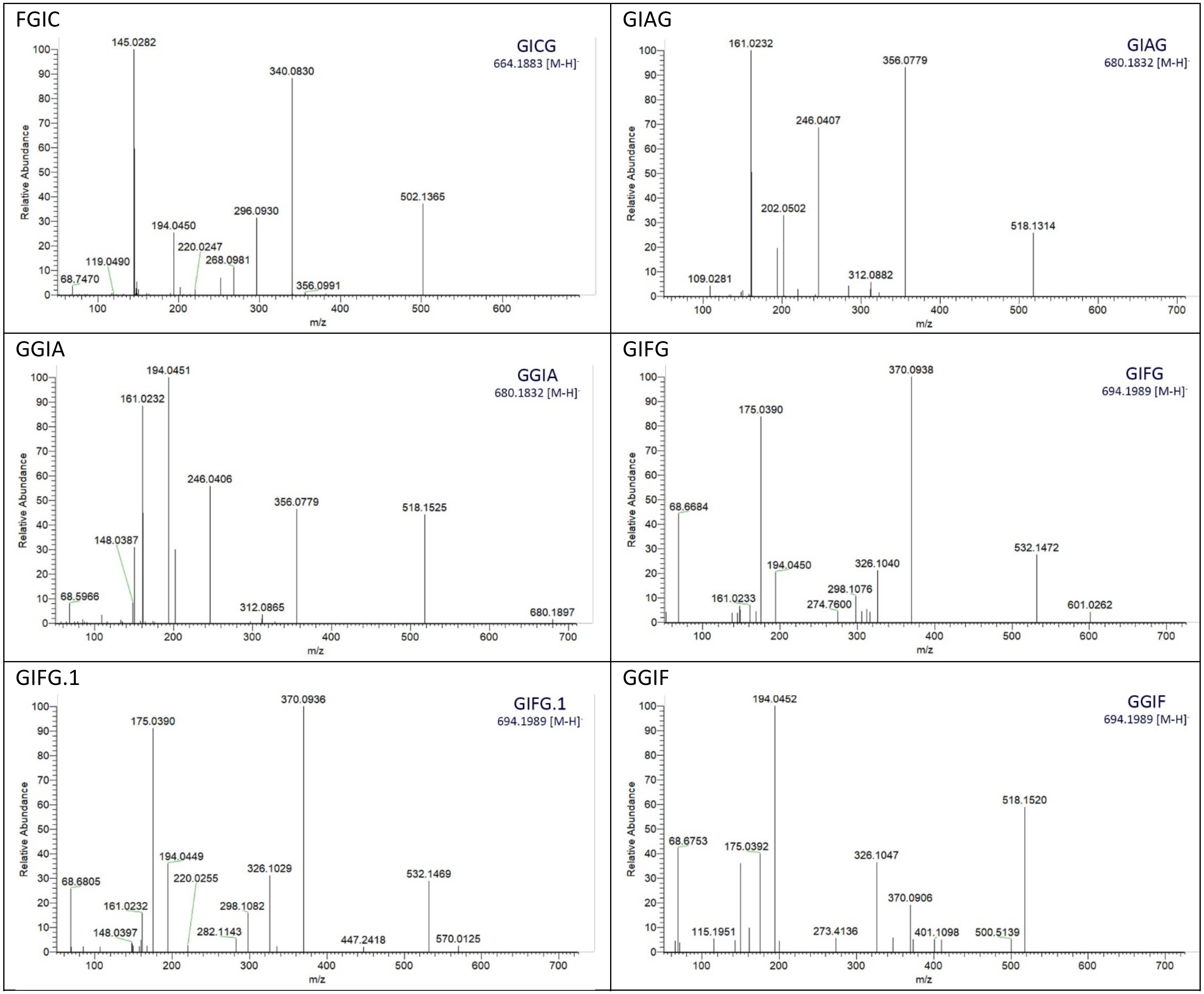

FGIC
GICG
664.1883 [M-H]-
145.0282
340.0830
502.1365
296.0930
194.0450
220.0247
268.0981
119.0490
68.7470
356.0991
GIAG
GIAG
680.1832 [M-H]-
161.0232
356.0779
246.0407
202.0502
518.1314
109.0281
312.0882
GGIA
GGIA
680.1832 [M-H]-
194.0451
161.0232
246.0406
356.0779
518.1525
148.0387
68.5966
312.0865
680.1897
GIFG
GIFG
694.1989 [M-H]-
370.0938
175.0390
68.6684
532.1472
194.0450
326.1040
298.1076
161.0233
274.7600
601.0262
GIFG.1
GIFG.1
694.1989 [M-H]-
370.0936
175.0390
194.0449
220.0255
326.1029
532.1469
68.6805
161.0232
298.1082
148.0397
282.1143
447.2418
570.0125
GGIF
GGIF
694.1989 [M-H]-
194.0452
518.1520
68.6753
175.0392
326.1047
370.0906
115.1951
273.4136
401.1098
500.5139
Relative Abundance
m/z

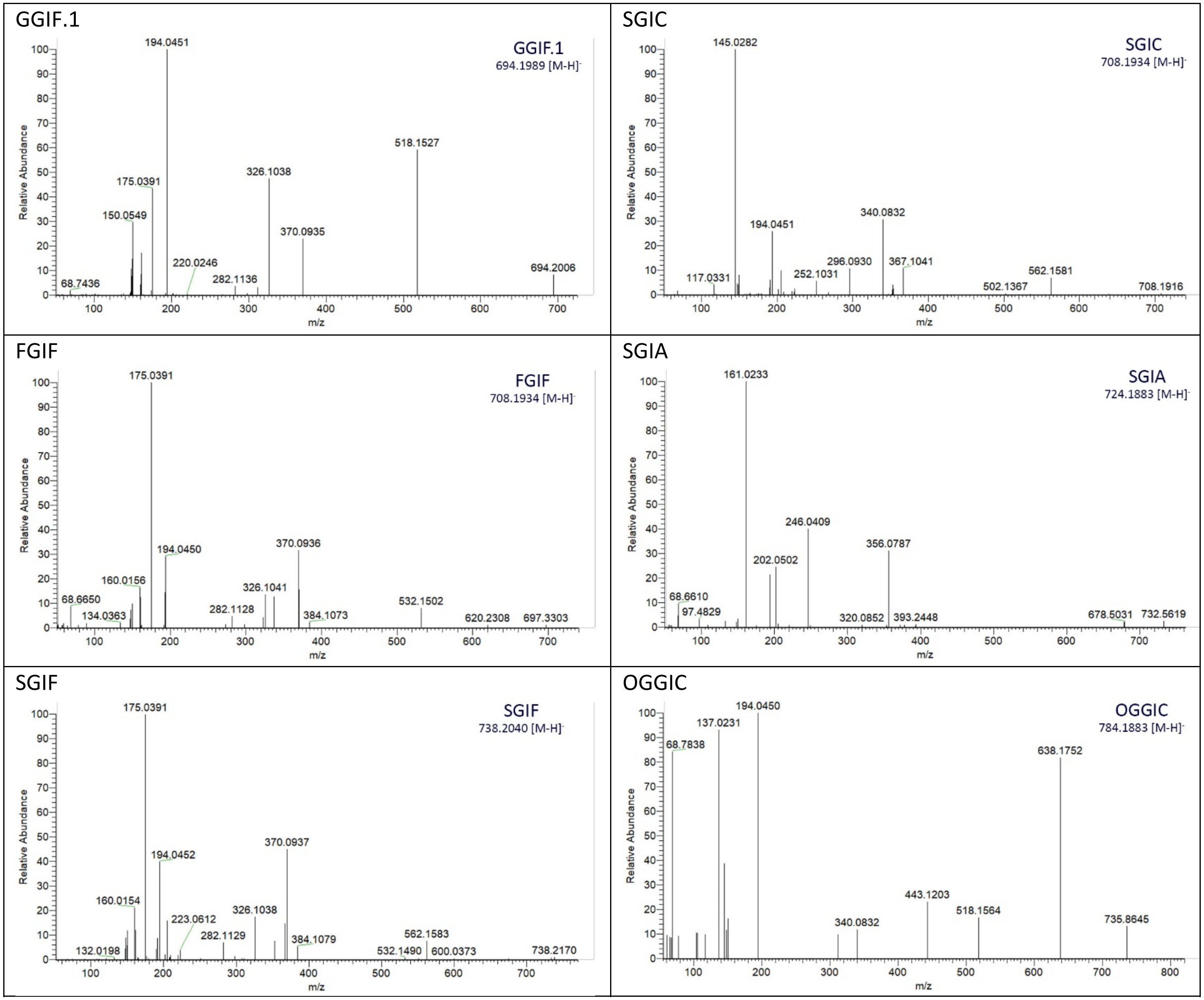

GGIF.1
GGIF.1
694.1989 [M-H]-
194.0451
518.1527
326.1038
175.0391
150.0549
370.0935
220.0246
282.1136
68.7436
694.2006
Relative Abundance
m/z
SGIC
SGIC
708.1934 [M-H]-
145.0282
340.0832
194.0451
117.0331
252.1031
296.0930
367.1041
502.1367
562.1581
708.1916
FGIF
FGIF
708.1934 [M-H]-
175.0391
194.0450
370.0936
160.0156
326.1041
68.6650
134.0363
282.1128
384.1073
532.1502
620.2308
697.3303
SGIA
SGIA
724.1883 [M-H]-
161.0233
246.0409
356.0787
202.0502
68.6610
97.4829
320.0852
393.2448
678.5031
732.5619
SGIF
SGIF
738.2040 [M-H]-
175.0391
370.0937
194.0452
160.0154
223.0612
326.1038
282.1129
384.1079
132.0198
562.1583
532.1490
600.0373
738.2170
OGGIC
OGGIC
784.1883 [M-H]-
194.0450
137.0231
68.7838
638.1752
443.1203
518.1564
340.0832
735.8645

CGGIC

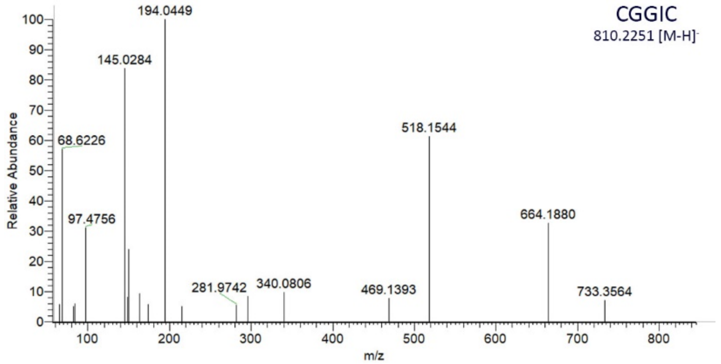

GGICG

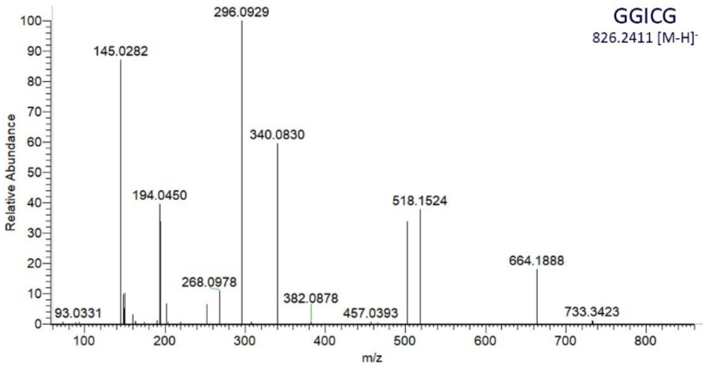

AGGIC

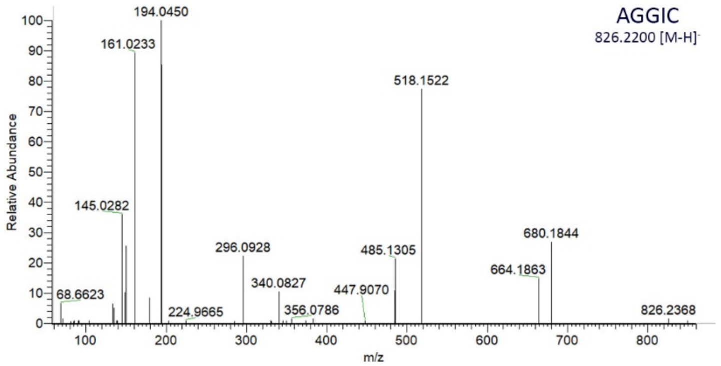

FGICG

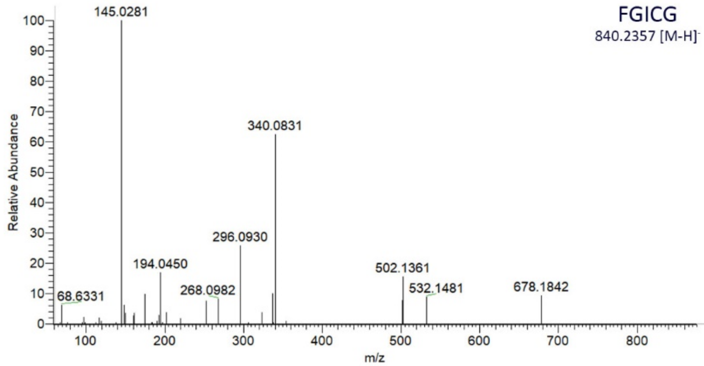

FGGIC

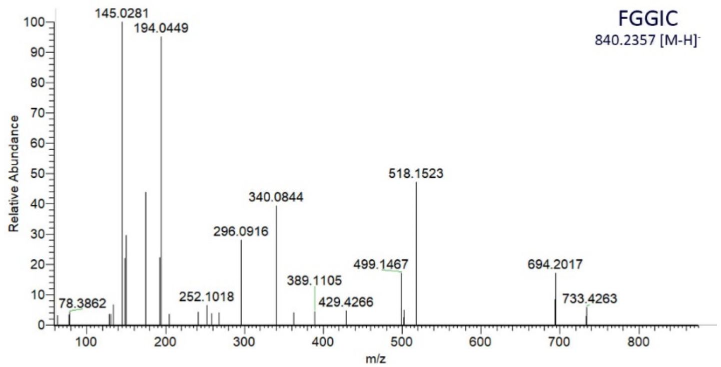

FGGIC.1

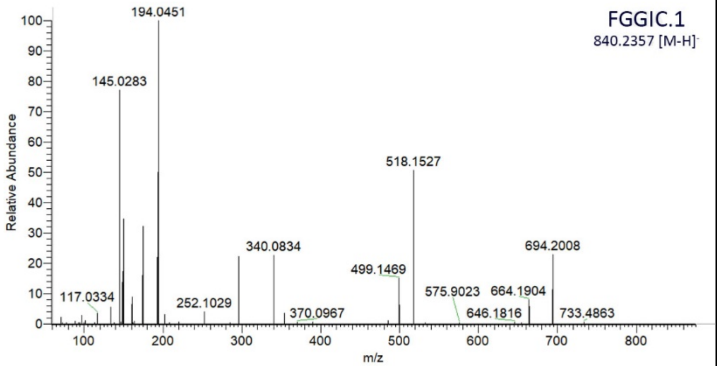

FGGIC.3

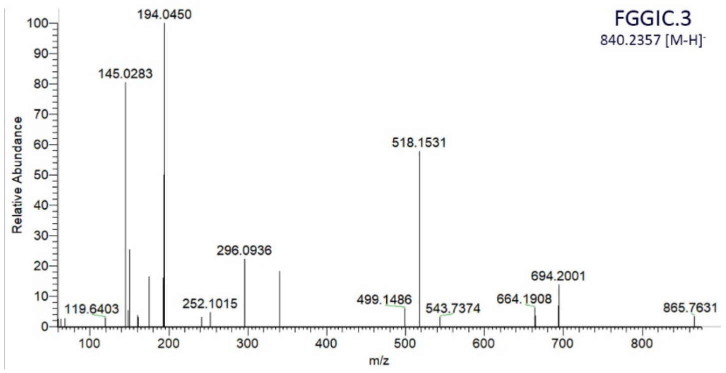

AGGIA

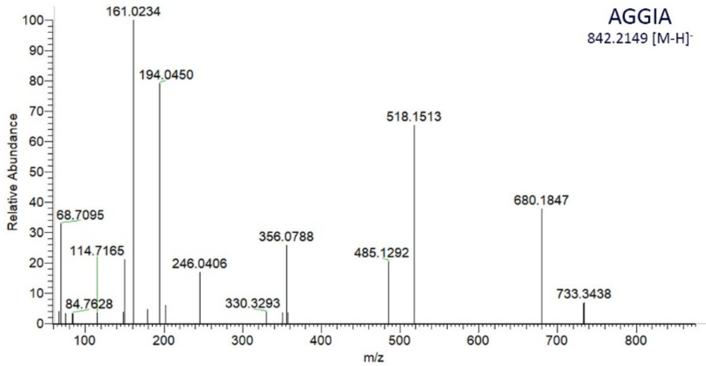

GGIAG

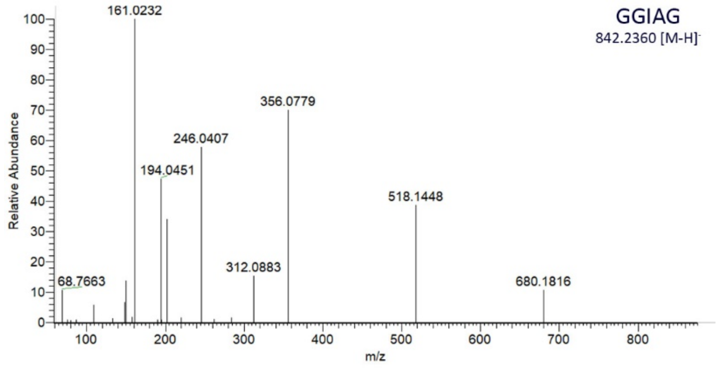

AGGIF

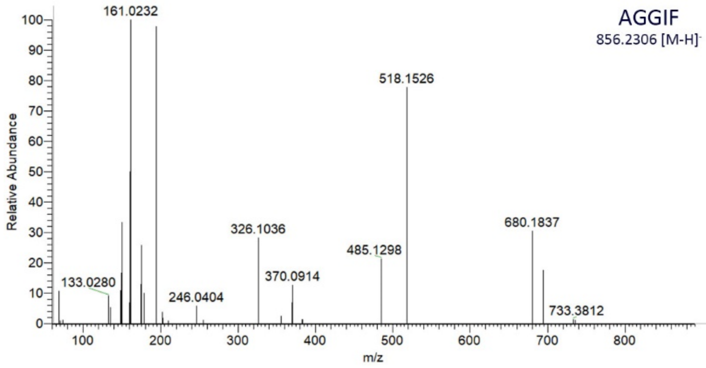

FGGIA

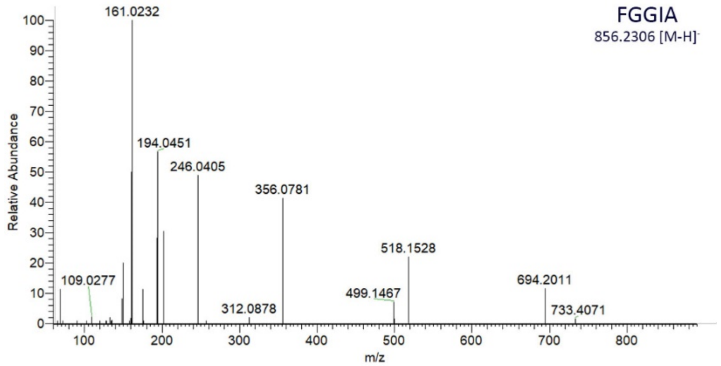

GGIFG

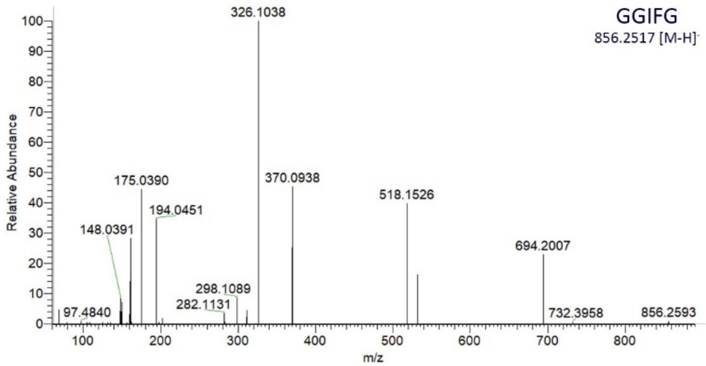

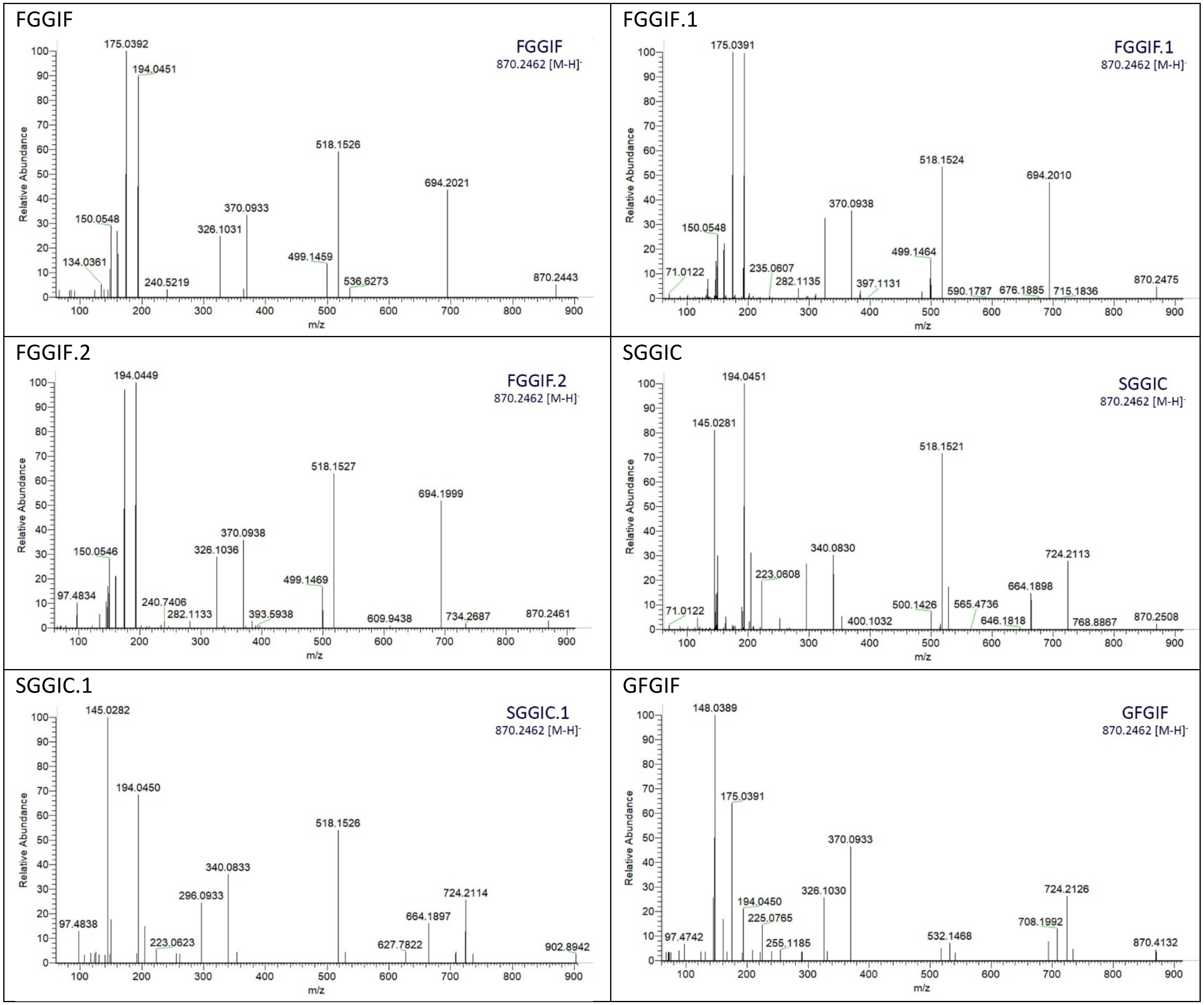
FGGIF
870.2462 [M-H]-
175.0392
194.0451
518.1526
694.2021
370.0933
326.1031
150.0548
134.0361
240.5219
499.1459
536.6273
870.2443
FGGIF.1
175.0391
518.1524
694.2010
370.0938
150.0548
499.1464
71.0122
235.0607
282.1135
397.1131
590.1787
676.1885
715.1836
870.2475
FGGIF.2
194.0449
518.1527
694.1999
370.0938
326.1036
150.0546
499.1469
97.4834
240.7406
282.1133
393.5938
609.9438
734.2687
870.2461
SGGIC
194.0451
145.0281
518.1521
340.0830
724.2113
223.0608
664.1898
71.0122
500.1426
565.4736
400.1032
646.1818
768.8867
870.2508
SGGIC.1
145.0282
194.0450
518.1526
340.0833
296.0933
724.2114
664.1897
97.4838
223.0623
627.7822
902.8942
GFGIF
148.0389
175.0391
370.0933
326.1030
724.2126
194.0450
225.0765
708.1992
97.4742
532.1468
255.1185
870.4132
Relative Abundance
m/z

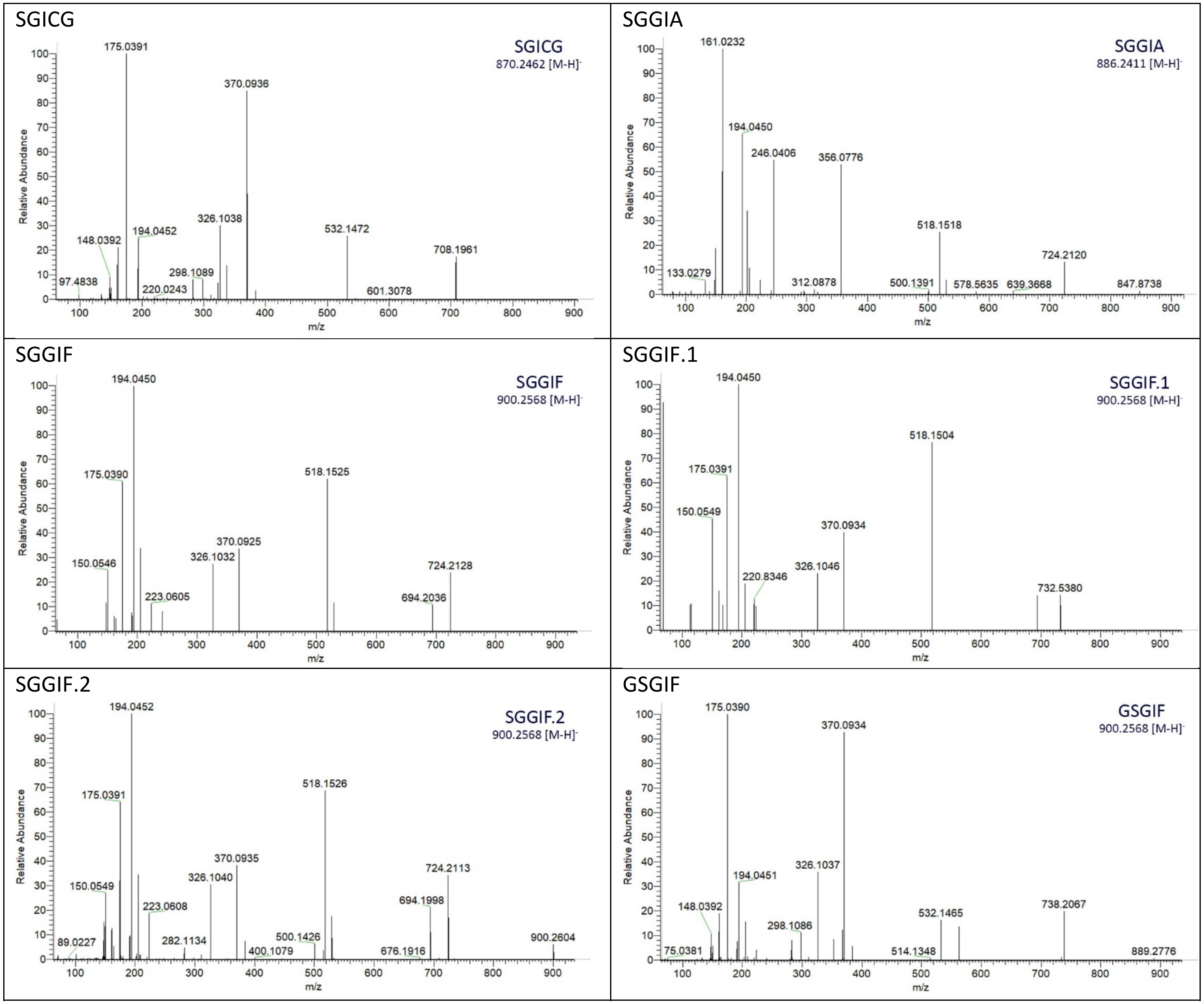
SGICG
SGICG
870.2462 [M-H]-
175.0391
370.0936
326.1038
532.1472
708.1961
148.0392
194.0452
298.1089
97.4838
220.0243
601.3078
Relative Abundance
m/z
SGGIA
SGGIA
886.2411 [M-H]-
161.0232
194.0450
246.0406
356.0776
518.1518
724.2120
133.0279
312.0878
500.1391
578.5635
639.3668
847.8738
Relative Abundance
m/z
SGGIF
SGGIF
900.2568 [M-H]-
194.0450
175.0390
518.1525
370.0925
326.1032
150.0546
724.2128
223.0605
694.2036
Relative Abundance
m/z
SGGIF.1
SGGIF.1
900.2568 [M-H]-
194.0450
518.1504
175.0391
150.0549
370.0934
326.1046
220.8346
732.5380
Relative Abundance
m/z
SGGIF.2
SGGIF.2
900.2568 [M-H]-
194.0452
518.1526
175.0391
370.0935
724.2113
326.1040
150.0549
694.1998
223.0608
500.1426
89.0227
282.1134
400.1079
676.1916
900.2604
Relative Abundance
m/z
GSGIF
GSGIF
900.2568 [M-H]-
175.0390
370.0934
326.1037
194.0451
148.0392
738.2067
532.1465
298.1086
75.0381
514.1348
889.2776
Relative Abundance
m/z

OGGICG

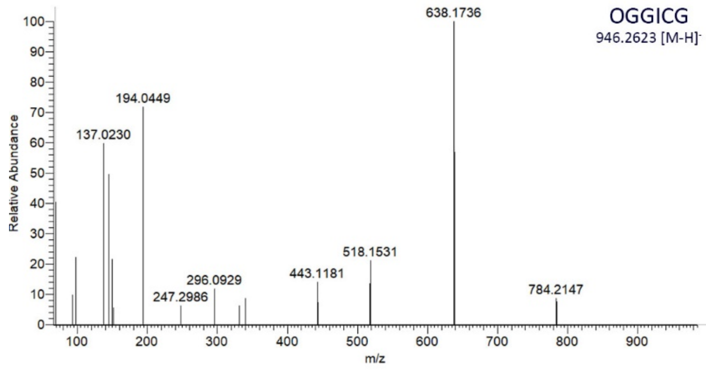

GAGGIC

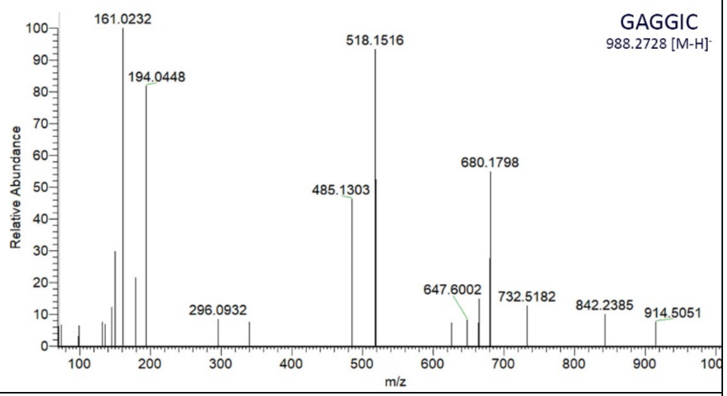

GAGGIC.1

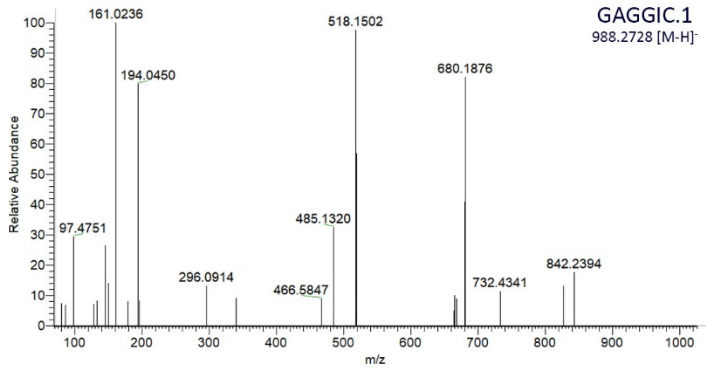

AGGGIC

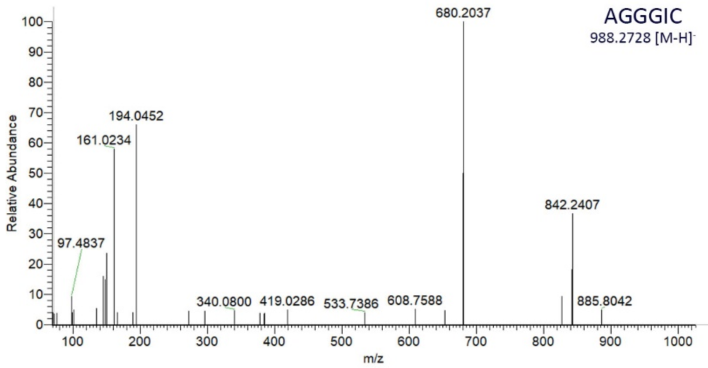

AGGICG

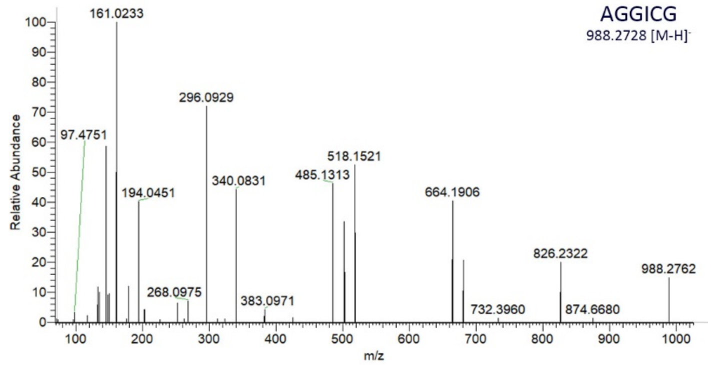

GGGICG

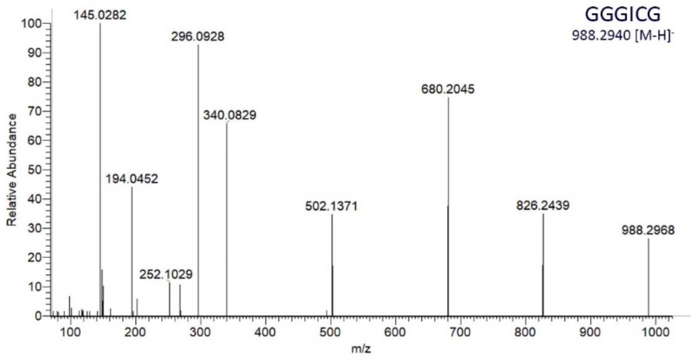

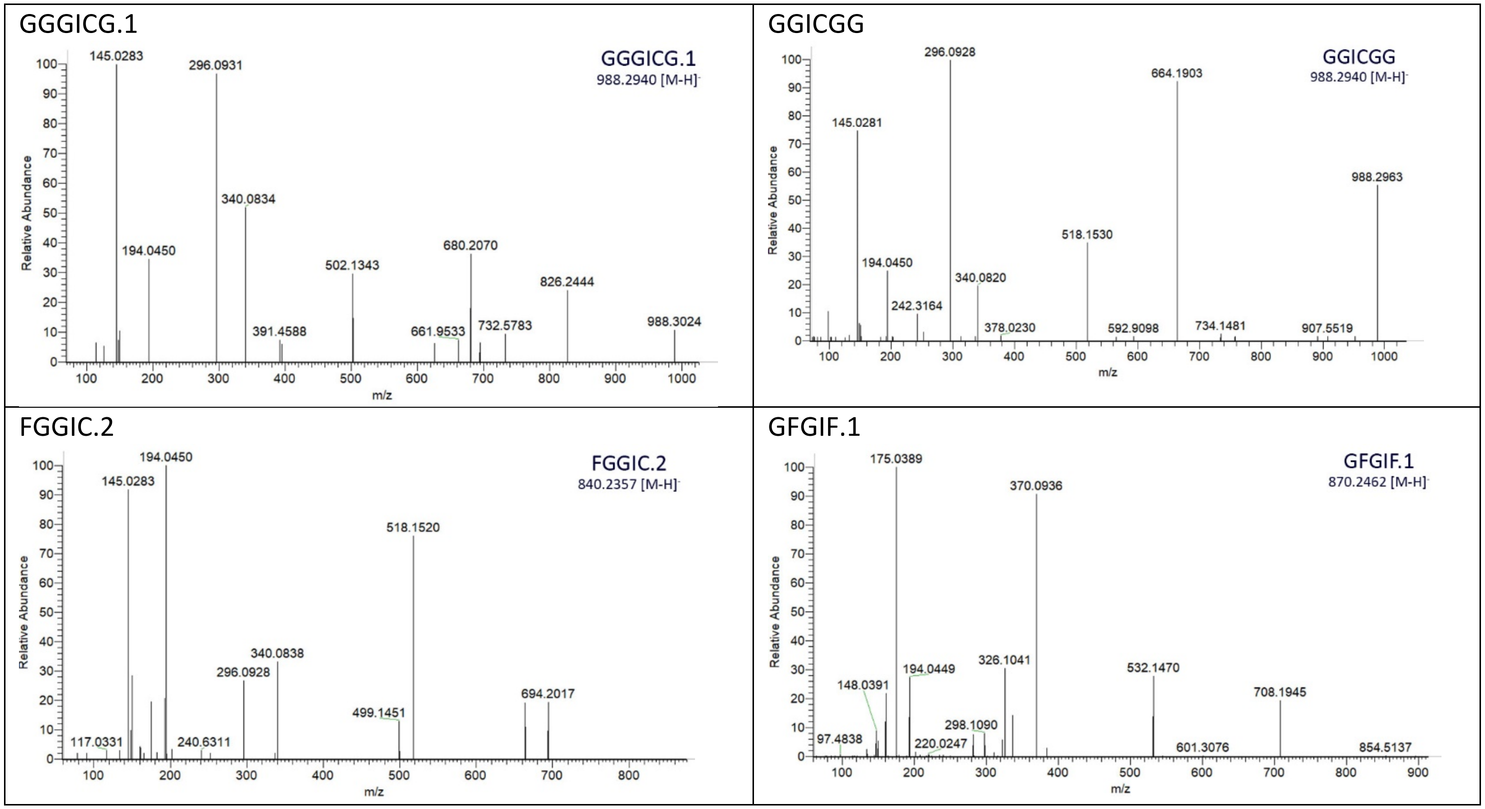
GGGICG.1
GGGICG.1
988.2940 [M-H]-
145.0283
296.0931
340.0834
194.0450
502.1343
680.2070
826.2444
391.4588
661.9533
732.5783
988.3024
Relative Abundance
m/z
GGICGG
GGICGG
988.2940 [M-H]-
296.0928
664.1903
145.0281
988.2963
518.1530
194.0450
340.0820
242.3164
378.0230
592.9098
734.1481
907.5519
Relative Abundance
m/z
FGGIC.2
FGGIC.2
840.2357 [M-H]-
194.0450
145.0283
518.1520
340.0838
296.0928
694.2017
499.1451
117.0331
240.6311
Relative Abundance
m/z
GFGIF.1
GFGIF.1
870.2462 [M-H]-
175.0389
370.0936
326.1041
532.1470
194.0449
148.0391
708.1945
298.1090
97.4838
220.0247
601.3076
854.5137
Relative Abundance
m/z
